# Supplementary material for: Ligand-displaying Escherichia coli cells and minicells for programmable delivery of toxic payloads via type IV secretion systems
Source: mBio. 2023 Sep 29;14(5):e02143-23. doi: 10.1128/mbio.02143-23 (PMC10653926; doi:10.1128/mbio.02143-23)
Supplement: Fig. S1 — Optimization of Nb/Ag pairing for pKM101 transfer. [file mbio.02143-23-s0001.pdf]

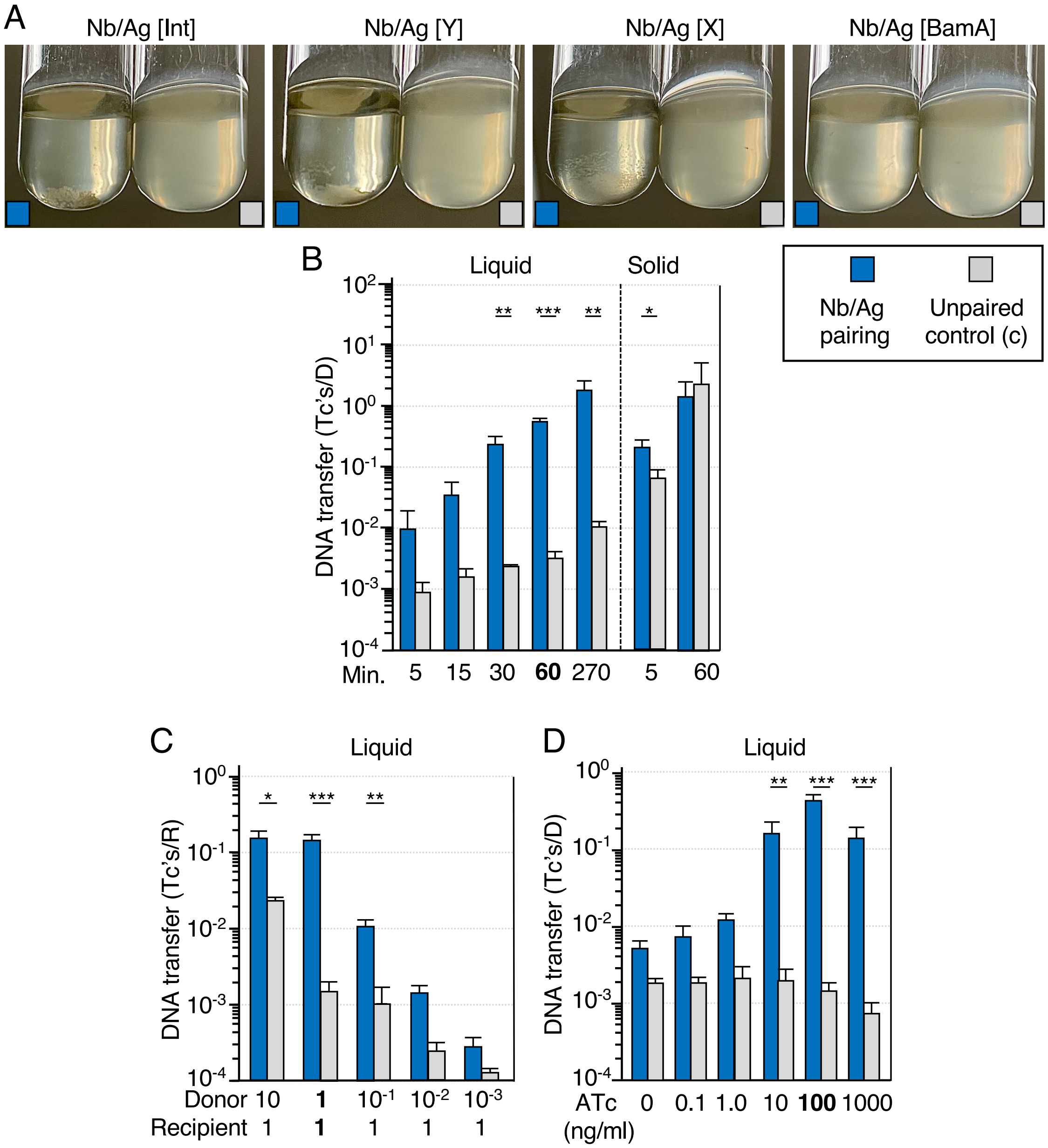


**Fig S1. Optimization of Nb/Ag pairing for pKM101 transfer. A.** Nb/Ag-mediated aggregation upon mixing of *E. coli* MC4100(pKM101) cells producing each of the Nb's shown and MC4100-Chl cells producing the cognate Ag or the null control. For Nb/Ag [BamA] pairing, the unpaired control in the aggregation assay (and for matings described below) was a mixture of MC4100(pKM101, p-null) and MC4100-Chl (naturally produces BamA). **B.** pKM101 transfer frequencies in liquid or solid-surface matings for the durations shown. Matings were repeated at least three times in triplicate, and the average transfer frequencies are presented as blue or gray bars with standard deviations shown as error bars. *p*-values between indicated data sets were calculated by the homoscedastic Student's *t*-test. * $p<0.05$, ** $p<0.001$, *** $p<0.0001$. **C.** Effects of varying MC4100(pKM101) donor colony-forming-units (CFUs) relative to those of the MC4100-Chl recipient (set at 1). Matings were carried out and analyzed as described for panel **B. D.** Effects of different anhydrotetracycline (ATc) inducer concentrations controlling expression of genes encoding the Nb, Ag, or null autotransporter proteins on pKM101 transfer in 60 min liquid matings. Donors produced Nb [Int] and recipients produced Ag [Int] or null control. Mating replicates and statistical analyses as described for panel **B.** Bold numbers in all panels: Optimized conditions for detection of the stimulatory effect of Nb/Ag [Int] pairing on pKM101 transfer. Further experiments evaluating Nb/Ag pairing on pKM101 self-transfer or mobilization were carried out with Nb/Ag [Int], 60 min liquid matings, a 1::1 donor::recipient seed ratio, and addition of ATc inducer to mating mixes at $10^2$ ng/ml (final conc.); these conditions are bolded in the associated panels.
