# Supplementary material for: Ligand-displaying Escherichia coli cells and minicells for programmable delivery of toxic payloads via type IV secretion systems
Source: mBio. 2023 Sep 29;14(5):e02143-23. doi: 10.1128/mbio.02143-23 (PMC10653926; doi:10.1128/mbio.02143-23)
Supplement: Fig. S2 — Optimization for F and RP4 transfer. [file mbio.02143-23-s0002.pdf]

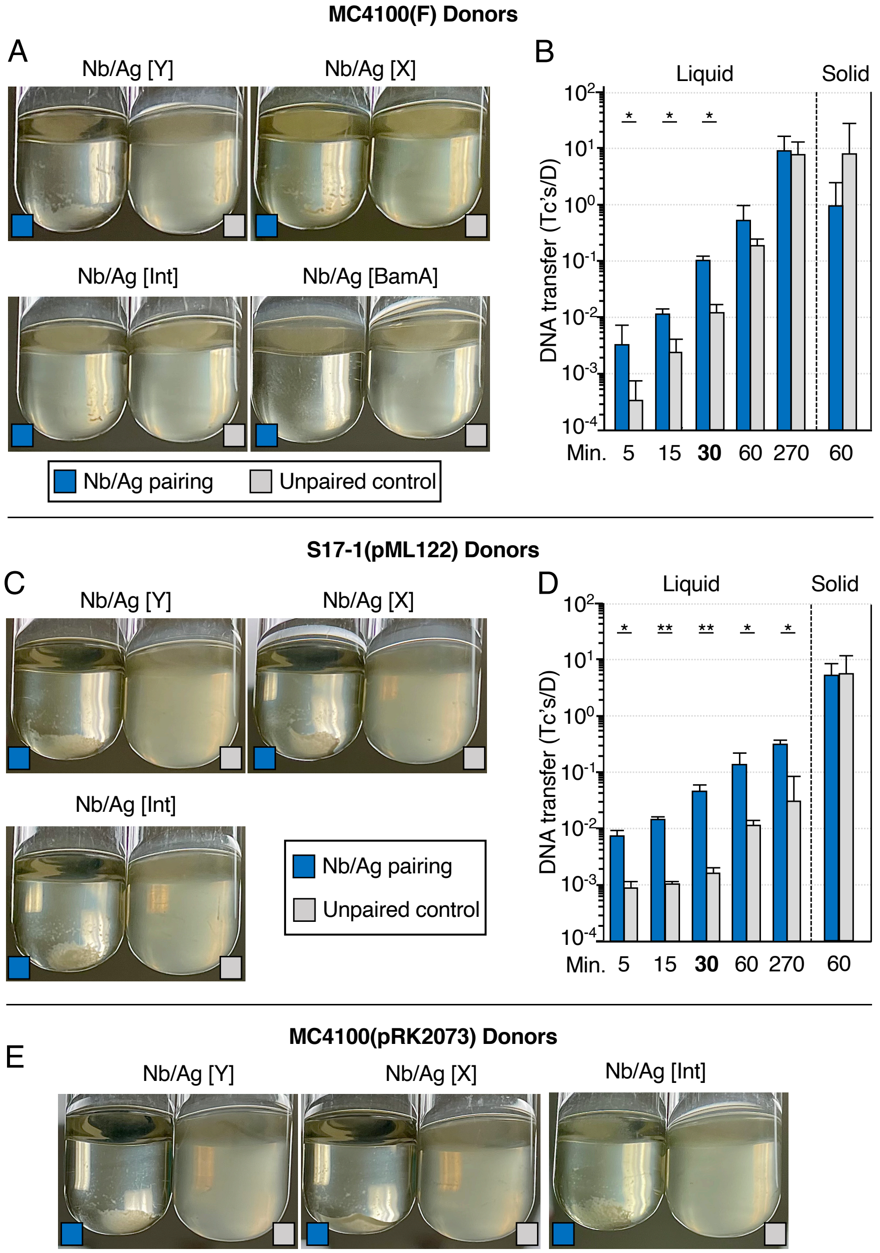

Fig. S2. Optimization of Nb/Ag pairing for F and RP4-mediated transfer. A. Nb/Ag-mediated aggregation upon mixing of *E. coli* MC4100(pOX38) (an F derivative; Table S1) cells producing each of the Nb's shown and MC4100-Chl cells producing the cognate Ag or the null control. Nb/Ag [BamA] pairing was analyzed as described in Fig. S1A. **B.** F transfer frequencies in liquid or solid-surface matings for the durations shown. **C.** Nb/Ag-mediated aggregation upon mixing of *E. coli* S17-1(pML122) (carries the IncP plasmid RP4 in the chromosome, Table S1) producing each of the Nb's shown and MC4100-Chl cells producing the cognate Ag or the null control. **D.** S17-1-mediated mobilization of pML22 through the RP4-encoded T4SS, with transfer frequencies in liquid or solid-surface matings for the durations indicated. **E.** Nb/Ag-mediated aggregation upon mixing of *E. coli* MC4100(pRK2073) (pRK2073 has the RP4 Tra and *oriT* regions, Table S1) producing each of the Nb's shown and MC4100-Chl cells producing the cognate Ag or the null control. Panels **B & D**: Matings were repeated at least three times in triplicate, and the average transfer frequencies are presented as blue or gray bars with standard deviations shown as error bars. *p*-values between indicated data sets were calculated by the homoscedastic Student's *t*-test. * $p<0.05$, ** $p<0.001$, *** $p<0.0001$. Further experiments evaluating Nb/Ag pairing on F and RP4-mediated transfer to *E. coli* recipients were carried out with Nb/Ag [Y], 30 min liquid matings (bolded in bar graphs), a 1::1 donor::recipient seed ratio, and addition of ATc inducer to mating mixes at 10^2 ng/ml (final conc.).
