# Supplementary material for: Ligand-displaying Escherichia coli cells and minicells for programmable delivery of toxic payloads via type IV secretion systems
Source: mBio. 2023 Sep 29;14(5):e02143-23. doi: 10.1128/mbio.02143-23 (PMC10653926; doi:10.1128/mbio.02143-23)
Supplement: Fig. S3 — Optimization for DNA transfer from E. coli to P. aeruginosa. [file mbio.02143-23-s0003.pdf]

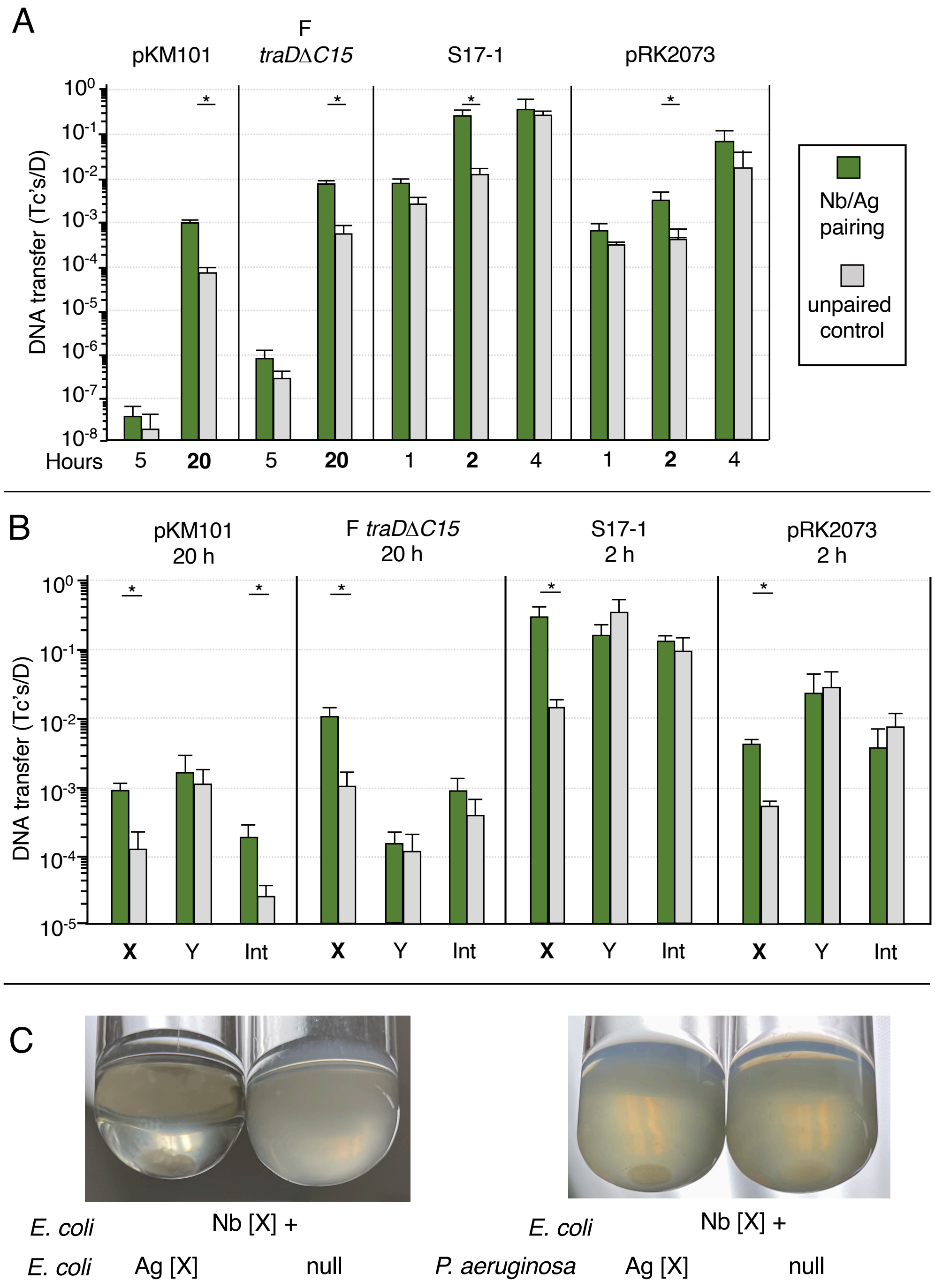

Fig. S3. Optimization of Nb/Ag pairing for DNA transfer from *E. coli* to *P. aeruginosa.* A. *E. coli* MC4100 or S17-1 donors producing Nb [X] and harboring the plasmids shown, plus the mobilizable plasmid pML122, were mated with *P. aeruginosa* PAO-1Δ*tssB1* recipients producing Ag [X] or null control on solid-surfaces for the durations indicated. Optimal mating durations for detection of Nb/Ag-enhanced transfer are in bold. **B.** Matings between *E. coli* donors and *P. aeruginosa* recipients as described above, except donors produced the Nb shown and recipients produced either the cognate Ag or the null control. Matings were carried out for the durations listed at the top. Matings were repeated at least three times in triplicate, and the average transfer frequencies are presented as green or gray bars with standard deviations shown as error bars. *p*-values between indicated data sets were calculated by the homoscedastic Student's *t*-test. * $p<0.05$, ** $p<0.001$, *** $p<0.0001$. **C.** Left: Nb/Ag-mediated aggregation upon mixing of *E. coli* YGLS1 producing chromosomally-encoded Nb [X] with YGLS2 producing chromosomally-encoded Ag [X] or YGLS3 producing chromosomally-encoded null control. Right: Nb/Ag-mediated aggregation upon mixing of *E. coli* YGLS1 producing chromosomally-encoded Nb [X] with *P. aeruginosa* PAO-1 producing Ag [X] or null from plasmids pYGL537 and pYGL542, respectively.
