# Supplementary material for: Ligand-displaying Escherichia coli cells and minicells for programmable delivery of toxic payloads via type IV secretion systems
Source: mBio. 2023 Sep 29;14(5):e02143-23. doi: 10.1128/mbio.02143-23 (PMC10653926; doi:10.1128/mbio.02143-23)
Supplement: Supplemental text — Supplemental Materials and Methods. [file mbio.02143-23-s0004.pdf]

Suppl. File 1. Supplemental Materials and Methods

Bacterial growth conditions. Strains were grown at 37°C with shaking in Lysogeny Broth (LB) or on solid LB agar. Strains or plasmids were maintained by selection with the following antibiotics (final concentrations and vendor sources in parantheses): carbenicillin (100 µg/ml; MP Biomedicals), spectinomycin (100 µg/ml; MP Biomedicals), chloramphenicol (20 µg/ml; Sigma), tetracycline (20 µg/ml; GoldBio), kanamycin (100 µg/ml for *E. coli*, 200 µg/ml for *P. aeruginosa*; Fisher), gentamycin (20 µg/ml for *E. coli*, 50 µg/ml for *P. aeruginosa*; CalBiochem), nalidixic acid (20 µg/ml for *E. coli*, 400 µg/ml for *P. aeruginosa*; Sigma), rifampicin (100 µg/ml; TCI).

Strains expressing surface-displayed adhesins from chromosome. *E. coli* MC4100 strains YGLS1, 2, and 3 respectively carry chromosomal copies of the autotransporter fusion genes *nb [X], ag [X],* and *null* along with their upstream anhydrotetracycline-inducible promoters. They were constructed by PCR amplification of the surface-displayed adhesin genes and *kanR* using the pDSG plasmids encoding Nb [X], Ag [X], or null as templates. The PCR products were then inserted into the *chlR* gene of MC4100-Chl by recombineering using pKD46 and selection for recombinants on LB plates containing kanamycin (100 µg/ml). Recombinants were verified by PCR amplification across the *chlR* gene and sequencing the amplicons.

Plasmid Constructions

Plasmids expressing tra genes: pYGL553 expressing P_{BAD}::$traN_{Strep}$ was constructed by PCR amplification of $traN_{Strep}$ using pOX38 as a template, digestion of the PCR fragment with NotI and HindIII, and ligation of the resulting product with similarly digested pBAD101. pYGL554 expressing P_{BAD}::$traN\Delta ED_{Strep}$ was constructed by deleting the TraN extracellular domain (ED) sequence from pYGL553 by inverse PCR. pYGL348 expressing P_{nahG}::*traDΔC15* was constructed by PCR amplification of *traDΔC15* using pOX38 as a template, digestion of the PCR fragment with NdeI and BamHI, and ligation of the resulting product with similarly digested pKG116.

Plasmids expressing surface-displayed adhesions: Plasmids pYGL536, 537, 539, 541, and 542 carry the broad-host-range pBBR origin of replication and genes encoding the intimin autotransporter β-barrel fused to Nb, Ag, or null passenger domains. pYGL536, 537, 539, 541, and 542 respectively code for surface-displayed Nb [X], Ag [X], Ag [Y], Ag [Int], and null. The plasmids were constructed by PCR amplification of the pBBR *oriV* and *rep* sequence using pBBR1MCS-2 as a template, digestion of the PCR fragment with XbaI and NheI, and ligation of the resulting product with similarly-digested pDSG plasmids listed in Table S1 that encode Nb [X], Ag [X], Ag [Y], Ag [Int], and null.

Plasmids expressing CRISPR/Cas9 system: pBBR-Cas9-Amp is derived from pCas9 and carries the broad-host-range pBBR origin of replication and an *ampR* gene. It was constructed by PCR amplification of the pBBR *oriV* and *rep* sequences using pBBR1MCS-2 as a template, and digestion of the PCR fragment with XbaI and XmaI. Next, *ampR* and its promoter was PCR amplified using pBAD24 as a template, and the PCR fragment was digested with XmaI and EagI. The two PCR products were ligated together with pCas9 digested with XbaI and EagI. pYGL533, 555, 562 are pBBR-Cas9-Amp plasmids carrying the origin of transfer (*oriT*) sequences of pKM101, pOX38, and RP4, respectively. They were constructed by PCR amplification of the *oriT* sequences using pKM101, pOX38, and S17-1 chromosome as templates, digestion of the PCR fragments with SalI, and ligation of the resulting products with similarly digested pBBR-Cas9-Amp.

pYGL535, pYGL557, and pYGL564 carry the two spacer sequences (designated B & C) that target the *chlR* gene of MC4100-Chl. They were constructed by annealing oligos Cat-gRNA-B/C_F and Cat-gRNA-B/C_R, and ligating the resulting product with BsaI-digested pYGL533, pYGL555, and pYGL562.

pYGL571 carries the two spacer sequences (designated B & C) that target the *tssJ1* gene of PAO-1. It was constructed by annealing oligos TssJ1-gRNA-B/C_F and TssJ1-gRNA-B/C_R, and ligating the resulting product with BsaI digested pYGL533. pYGL575 and pYGL579 are pML122 plasmids harboring the CRISPR/Cas9 sequences bearing the *tssJ1* targeting sequences and gRNA- control sequence, respectively. They were derived by

digestion of pYGL571 and pYGL533 with XbaI and XmaI to isolate the CRISPR/Cas9 *tssJ1* and gRNA- control elements. Next, the XbaI/XmaI fragments carrying the CRISPR/Cas9 elements were ligated to PCR-amplified, linearized pML122 by Gibson assembly.

Plasmids expressing fluorescent proteins: pBAD24-mCerulean3 carries *mCerulean3* and was constructed by PCR amplification of *mCerulean3* using pmCer3 (gift from Margolin lab) as a template, digestion of the PCR fragment with NheI and HindIII, and ligation of the resulting product with similarly digested pBAD24. pBAD24-mCherry carries *mCherry* and was constructed by PCR amplification of *mCherry* using pRSET-BmCherry (Addgene Plasmid #108857) as a template, PCR amplification of the plasmid vector using pBAD24 as a template, and ligation of both PCR fragments by FastCloning.
