# Supplementary material for: Ligand-displaying Escherichia coli cells and minicells for programmable delivery of toxic payloads via type IV secretion systems
Source: mBio. 2023 Sep 29;14(5):e02143-23. doi: 10.1128/mbio.02143-23 (PMC10653926; doi:10.1128/mbio.02143-23)
Supplement: Table S1 — Strains and plasmids used in this study. [file mbio.02143-23-s0005.pdf]

Table S1. Strains and plasmids used in this study.

Strains or plasmids	Relevant characteristics	Source
**Strains**		
E.coli		
MC4100	F^- [*araD139*]$_{B/r}$ Δ(*argF-lac*)*169* $λ^-$ *e14*$^-$ *flhD5301* *Δ(fruK-yeiR)725(fruA25) relA1 rpsL150(strR) rbsR22 Δ(fimB-fimE)632(::IS1) deoC*	Lab stock
MC4100-Rif	MC4100 mutated to rifampin (*rifR*) resistance	This study
MC4100-Chl	MC4100::*chlR*	Gift from Konovalova lab
DH5α-Rif	DH5α mutated to *rifR*	This study
AA116	MC4100 *rifR chlR*	(1)
S17-1	*pro, res*$^-$ *hsdR17 (rK*$^-$ *mK*$^+$*) recA*$^-$ with an integrated *RP4-2-Tc::Mu-Km::Tn7*	(2)
WM3886 (equivalent to CGSC#6397)	*minD.* Minicell producing strain	Gift from Margolin lab; originally from *E. coli* Genetic Stock Center, Yale Univ.
WM3886-Nal	WM3886 mutated to *nalR*	This study
YGLS1	MC4100 with *nb[X] kanR* cassette inserted into *chlR*	This study
YGLS2	MC4100 with *ag[X] kanR* cassette inserted into *chlR*	This study
YGLS3	MC4100 with *null kanR* cassette inserted into *chlR*	This study
P. aeruginosa		
PAO-1Δ*tssB1*	Δ*tssB1*; T6SS-	*Pseudomonas* Transposon Mutant Collection, University of Washington Genome Sciences
**Plasmids**		
pBAD101	SpcR; pSC101 with P_{BAD} promoter	(3)
pBAD24	CrbR; ColE1 with P_{BAD} promoter	(4)
pKG116	ChlR; pACYC184 with P_{nahG} promoter	(5)
pBBR1MCS-2	KanR; broad-host-range pBBR mobilizable shuttle and expression vector	(6)
pCas9	ChlR; bacterial expression of Cas9 nuclease, tracrRNA and crRNA guide	Addgene Plasmid #42876 (7)
pKM101	SpcR; IncN conjugative plasmid	(8)
pOX38::Tc	TetR; Tra^+ F plasmid derivative	(9)
pRK2073	StrpR; ColE1 replicon with IncP RP4 conjugative transfer genes	(10)
pML122	GenR; mobilizable RSF1010 derivative	(11)
pKM101Δ*traJ*	SpcR; pKM101 deleted of *traJ*	(3)
pKM101Δ*traM* (also pJG1002)	SpcR; pKM101 deleted of *traM*	(12)
pCGR125	CrbR; pBAD24 with pKM101 *pep-traG* Tra region	(13)
pCGR107	CrbR; pCGR125 deleted of *traC*	(13)
pCGR135	CrbR; pCGR125 deleted of *pep*	(13)
pJG142	SpcR; pSC101 with pKM101 *oriT-traI mob* region	(12)

pOX38Δ*traN*	TetR; pOX38 deleted of *traN*	(14)
pOX38Δ*traD*	TetR; pOX38 deleted of *traD*	(15)
pOX38Δ*traA*	TetR; pOX38 deleted of *traA*	(14)
pYGL22	CrbR; pBAD24 with P_{BAD}::$traJ_{Strep}$	(3)
pDSG372 (p-Nb [X])	KanR; p15A producing surface-displayed Nb [X]	(16)
pDSG398 (p-Nb [Y])	KanR; p15A producing surface-displayed Nb [Y]	(16)
pNb [Int]	KanR; p15A producing surface-displayed Nb [Int]	(17)
pNb [BamA]	KanR; p15A producing surface-displayed Nb [BamA]	(17)
pDSG358 (p-Ag [X])	KanR; p15A producing surface-displayed Ag [X]	(16)
pDSG360 (p-Ag [Y])	KanR; p15A producing surface-displayed Ag [Y]	(16)
pAg [Int]	KanR; p15A producing surface-displayed Intimin	(17)
pDSG323 (p-null)	KanR; p15A producing β-barrel of intimin autotransporter but no passenger domain/adhesion	(16)
pYGL348	ChlR; pKG116 with P_{nahG}::$_{Strep}traD\Delta C15$ (pOX38)	This study
pYGL553	SpcR; pBAD101 with P_{BAD}::$traN_{Strep}$ (pOX38)	This study
pYGL554	SpcR; pBAD101 with P_{BAD}::$traN\Delta ED_{Strep}$ (pOX38)	This study
pYGL536	KanR; pBBR, produces surface-displayed Nb [X]	This study
pYGL537	KanR; pBBR, produces surface-displayed Ag [X]	This study
pYGL539	KanR; pBBR, produces surface-displayed Ag [Y]	This study
pYGL541	KanR; pBBR, produces surface-displayed Ag[Int]	This study
pYGL542	KanR; pBBR, produces β-barrel of intimin autotransporter but no passenger domain/adhesion	This study
pYGL533	AmpR; pBBR with pKM101 *oriT* sequence and CRISPR/Cas9 gRNA-	This study
pYGL535	AmpR; pBBR with pKM101 *oriT* sequence and CRISPR/Cas9 $gRNA_{chlR}$	This study
pYGL555	AmpR; pBBR with pOX38 *oriT* sequence and CRISPR/Cas9 gRNA-	This study
pYGL557	AmpR; pBBR with pOX38 *oriT* sequence and CRISPR/Cas9 $gRNA_{chlR}$	This study
pYGL562	AmpR; pBBR with RP4 *oriT* sequence and CRISPR/Cas9 gRNA-	This study
pYGL564	AmpR; pBBR with RP4 *oriT* sequence and CRISPR/Cas9 $gRNA_{chlR}$	This study
pYGL571	AmpR; pBBR with pKM101 *oriT* sequence and CRISPR/Cas9 $gRNA_{tssJ1}$	This study
pYGL575	GenR; pML122 with CRISPR/Cas9 $gRNA_{tssJ1}$	This study
pYGL579	GenR; pML122 with CRISPR/Cas9 gRNA-	This study
pBAD24-mCerulean3	CrbR; pBAD24 with P_{BAD}::*mCerulean3*	This study; derived from pmCer3 originally from (18)
pBAD24-mCherry	CrbR; pBAD24 with P_{BAD}::*mCherry*	This study
pKD46	AmpR; temperature sensitive plasmid for λRED recombineering in *E. coli*	(19)

References:

1. Al Mamun AAM, Kishida K, Christie PJ. 2021. Protein transfer through an F plasmid-encoded type IV secretion system suppresses the mating-induced SOS response. mBio 12:e0162921. doi: 10.1128/mBio.01629-21.
2. Simon R, Priefer U, Puhler A. 1983. A broad host range mobilization system for *in vivo* genetic engineering: transposon mutagenesis in Gram negative bacteria. Bio/Technology 1:37-45.
3. Li YG, Christie PJ. 2020. The TraK accessory factor activates substrate transfer through the pKM101 type IV secretion system independently of its role in relaxosome assembly. Mol Microbiol 114:214-229.
4. Guzman LM, Belin D, Carson MJ, Beckwith J. 1995. Tight regulation, modulation, and high-level expression by vectors containing the arabinose P_{BAD} promoter. J Bacteriol 177:4121-30.
5. Chang AC, Cohen SN. 1978. Construction and characterization of amplifiable multicopy DNA cloning vehicles derived from the P15A cryptic miniplasmid. J Bacteriol 134:1141-56.
6. Kovach ME, Phillips RW, Elzer PH, Roop RM, 2nd, Peterson KM. 1994. pBBR1MCS: a broad-host-range cloning vector. Biotechniques 16:800-2.
7. Jiang W, Bikard D, Cox D, Zhang F, Marraffini LA. 2013. RNA-guided editing of bacterial genomes using CRISPR-Cas systems. Nat Biotechnol 31:233-9.
8. Whitaker N, Berry TM, Rosenthal N, Gordon JE, Gonzalez-Rivera C, Sheehan KB, Truchan HK, VieBrock L, Newton IL, Carlyon JA, Christie PJ. 2016. Chimeric coupling proteins mediate transfer of heterologous type IV effectors through the *Escherichia coli* pKM101-encoded conjugation machine. J Bacteriol 198:2701-18.
9. Anthony KG, C. Sherburne, R. Sherburne, and L. S. Frost. 1994. The role of the pilus in recipient cell recognition during bacterial conjugation mediated by F-like plasmids. Mol Microbiol 13:939-953.
10. Leong SA, Ditta GS, Helinski DR. 1982. Heme biosynthesis in *Rhizobium*. Identification of a cloned gene coding for delta-aminolevulinic acid synthetase from *Rhizobium meliloti*. J Biol Chem 257:8724-30.
11. Fullner KJ, Lara JC, Nester EW. 1996. Pilus assembly by *Agrobacterium* T-DNA transfer genes. Science 273:1107-9.
12. Gordon JE, Costa TRD, Patel RS, Gonzalez-Rivera C, Sarkar MK, Orlova EV, Waksman G, Christie PJ. 2017. Use of chimeric type IV secretion systems to define contributions of outer membrane subassemblies for contact-dependent translocation. Mol Microbiol 105:273-293.
13. Gonzalez-Rivera C, Khara P, Awad D, Patel R, Li YG, Bogisch M, Christie PJ. 2019. Two pKM101-encoded proteins, the pilus-tip protein TraC and Pep, assemble on the *Escherichia coli* cell surface as adhesins required for efficient conjugative DNA transfer. Mol Microbiol 111:96-117.
14. Kishida K, Bosserman RE, Harb L, Khara P, Song L, Hu B, Zeng L, Christie PJ. 2022. Contributions of F-specific subunits to the F plasmid-encoded type IV secretion system and F pilus. Mol Microbiol 117:1275-1290.
15. Harb L, Chamakura K, Khara P, Christie PJ, Young R, Zeng L. 2020. ssRNA phage penetration triggers detachment of the F-pilus. Proc Natl Acad Sci U S A 117:25751-25758.
16. Glass DS, Riedel-Kruse IH. 2018. A synthetic bacterial cell-cell adhesion toolbox for programming multicellular morphologies and patterns. Cell 174:649-658 e16.
17. Ting SY, Martinez-Garcia E, Huang S, Bertolli SK, Kelly KA, Cutler KJ, Su ED, Zhi H, Tang Q, Radey MC, Raffatellu M, Peterson SB, de Lorenzo V, Mougous JD. 2020. Targeted depletion of bacteria from mixed populations by programmable adhesion with antagonistic competitor cells. Cell Host Microbe 28:313-321 e6.
18. Markwardt ML, Kremers GJ, Kraft CA, Ray K, Cranfill PJ, Wilson KA, Day RN, Wachter RM, Davidson MW, Rizzo MA. 2011. An improved cerulean fluorescent protein with enhanced brightness and reduced reversible photoswitching. PLoS One 6:e17896.
19. Datsenko KA, Wanner BL. 2000. One-step inactivation of chromosomal genes in *Escherichia coli* K-12 using PCR products. Proc Natl Acad Sci U S A 97:6640-5.
