# Supplementary material for: Ligand-displaying Escherichia coli cells and minicells for programmable delivery of toxic payloads via type IV secretion systems
Source: mBio. 2023 Sep 29;14(5):e02143-23. doi: 10.1128/mbio.02143-23 (PMC10653926; doi:10.1128/mbio.02143-23)
Supplement: Table S2 — Oligonucleotides used in this study. [file mbio.02143-23-s0006.pdf]

**Table S2. Oligonucleotides used in this study.**

| Oligo name | Sequence (5' to 3') | Purpose |
|---|---|---|
| For plasmid construction | | |
| TraNΔED-OX_F | [phos]AAAAAAGCCTCGTACACCCCGTATGTTG | pYGL554 construction |
| TraNΔED-OX_R | [phos]CCATGTGGTACTCCCCTGCAGC | pYGL554 construction |
| NotI-TraN_F | ATATATGCGGCCGCAGGAGGAATTCACCATGAAACGTATTTTACCTC | pYGL553, and 554 constructions |
| HindIII-TraN_R | ATATATAAGCTTTTACTTTTCGAATTGAGGATGAGACCACTGTC | pYGL553, and 554 constructions |
| NdeI-strep-TraD_F | ATATATATCATATGTGGTCTCATCCTCAATTCGAAAAGGGTACCAGTTTTAACGCAAAGGATATGACCCAGGGC | pYGL348 construction |
| BamHI-TraD_R | ATATATATGGATCCTCAATTAATGTTCACCTCTTCACGACGCTGCATC | pYGL348 construction |
| XbaI-BBR_F | ATATATTCTAGACCGTGGATATGTGGACGATGGCC | pYGL533, 535, 536, 537, 539, 541, 542, 555, 557, 562, and 564 constructions |
| NheI-BBR_R | ATATATGCTAGCGGGTTGCTGCGCAACCCAAGTG | pYGL536, 537, 539, 541, and 542 constructions |
| XmaI-BBR_R | ATATATCCCGGGGGGTTGCTGCGCAACCCAAGTG | pYGL533, 535, 555, 557, 562, and 564 constructions |
| XmaI_Amp+p_F | ATATATCCCGGGCTAAATACATTCAAATATGTATCCGCTCATGAGAC | pYGL533, 535, 555, 557, 562, and 564 constructions |
| EagI_Amp+p_R | ATATATCGGCCGCTTGGTCTGACAGTTACCAATGCTTAATCAG | pYGL533, 535, 555, 557, 562, and 564 constructions |
| SalI-pKM101-oriT_F | ATATATGTCGACGTACCCTCATTTAGAATGATGTAATTTTGATGTATTTCTG | pYGL533, and 535 constructions |
| SalI-pKM101-oriT_R | ATATATGTCGACCAGTCCTCACATTGTGCATTTCTTAAACAAAAG | pYGL533, and 535 constructions |
| SalI-pOX38-oriT_F | ATATATGTCGACAATCTACCTGCATCAGTCCGCTGCC | pYGL555, and 557 constructions |
| SalI-pOX38-oriT_R | ATATATGTCGACGCTGATATACAGGTTCACCTTAGCCATTAGAG | pYGL555, and 557 constructions |
| SalI-RP4-oriT_F | ATATATGTCGACGAATAAGGGACAGTGAAGAAGGAACACCC | pYGL562, and 564 constructions |
| SalI-RP4-oriT_R | ATATATGTCGACTGTAGACTTTCCTTGGTGTATCCAACGGC | pYGL562, and 564 constructions |
| Cat-gRNA-B/C_F | AAACAATGAAAGACGGTGAGCTGGTGATAGTTTTAGAGCTATGCTGTTTTGAATGGTCCCAAAACTATATTCGCAAGATGTGGCGTGTTAG | pYGL535, 557, and 564 constructions |
| Cat-gRNA-B/C_R | AAAACTAACACGCCACATCTTGCGAATATAGTTTTGGGACCATTCAAAACAGCATAGCTCTAAAACTATCACCAGCTCACCGTCTTTCATT | pYGL535, 557, and 564 constructions |
| TssJ1-gRNA-B/C_F | AAACCCTCACCGACAACGCCCAGTCGACCCTGTTTTAGAGCTATGCTGTTTTGAATGGTCCCAAAACGCCTACCGCGACCTCGACCGCGCCACCG | pYGL575 construction |
| TssJ1-gRNA-B/C_R | AAAACGGTGGCGCGGTCGAGGTCGCGGTAGGCGTTTTGGGACCATTCAAAACAGCATAGCTCTAAAACAGGGTCGACTGGGCGTTGTCGGTGAGG | pYGL575 construction |

| | | |
|---|---|---|
| GA-pML122-XbaIXmaI_F | GAGATAAATTGCACTGAAATCATGCCTTATG GAACTCCTCGATCC | pYGL575, and 579 constructions |
| GA-pML122-XbaIXmaI_R | CATATTTGAATGTATTTAGCCTCATGATGTCT AACGGCCAAGGTAAG | pYGL575, and 579 constructions |
| KKTX607-NheI-mCeru_F | AGAGAGGCTAGCAGGAGGAATTCACCATGG TGAGCAAGGGCGAGGAGCTG | pBAD24-mCerulean3 construction |
| KKTX608-HindIII-mCeru_R | AGAGAGAAGCTTTTACTTGTACAGCTCGTCC | pBAD24-mCerulean3 construction |
| Orb167-mCherry_F | GCAGGAGGAATTCACCATGGTGAGCAAGGG CGAGGA | pBAD24-mCherry construction |
| Orb168-mCherry_R | CAAAACAGCCAAGCTTTTACTTGTACAGCTC GTCCATGCC | pBAD24-mCherry construction |
| pBAD24-FC_F | AAGCTTGGCTGTTTTGGCGGATGAG | pBAD24-mCherry construction |
| pBAD24-FC_R | CATGGTGAATTCCTCCTGCTAGCC | pBAD24-mCherry construction |
| For plasmid sequencing | | |
| pKG116_SeqF | CTCGCTTGGGTTATTGCTGGTG | pYGL348 sequencing |
| pKG116_SeqR | GCGACAAGTTGCTGCGATTCTC | pYGL348 sequencing |
| pBAD24_SeqF | ATGCCATAGCATTTTTATCC | pYGL553, pYGL554, pBAD24-mCerulean3, and pBAD24-mCherry sequencing |
| pBAD24_SeqR | GATTTAATCTGTATCAGG | pYGL321 sequencing |
| pBAD101_SeqR | CAGGTCATTCAGACTGGCTAATG | pYGL553, and pYGL554 sequencing |
| pBBR_SeqF | CGCAACGCATAATTGTTGTCGCG | pYGL536, pYGL537, pYGL539, pYGL541, pYGL542, pYGL533, pYGL535, pYGL555, pYGL557, pYGL562, and pYGL564 sequencing |
| pBBR_SeqR | GCTTGTTCTTGGCTTGAATGCCG | pYGL536, pYGL537, pYGL539, pYGL541, pYGL542, pYGL533, pYGL535, pYGL555, pYGL557, pYGL562, and pYGL564 sequencing |
| gRNA_SeqF | GAGTCAGCTAGGAGGTGACTGAA | pYGL533, pYGL535, pYGL555, pYGL557, pYGL562, pYGL564, pYGL575, and pYGL579 sequencing |
| For strain construction | | |
| Cm-NbAg_F | AATGGCATCGTAAAGAACATTTTGAGGCATT TCAGTCAGTTGCTCAATGTCCTGACGTCTAA GAAACCATTATTATCATGACATTAACC | YGLS1, 2, 3 constructions |
| Cm-NbAg_R | TAATTCATTAAGCATTCTGCCGACATGGAAG CCATCACAGACGGCATGATCTCGTGAAGAAG GTGTTGCTGACTCATACC | YGLS1, 2, 3 constructions |
| For strain verification | | |
| Cm_F | TCACTGGATATACCACCGTTGATATATCCC | YGLS1, 2, 3 verification |
| Cm_R | CCTGCCACTCATCGCAGTACTGTTG | YGLS1, 2, 3 verification |
